# Unveiling GPT-4V's hidden challenges behind high accuracy on USMLE questions: Observational Study

**DOI:** 10.2196/65146

**Published:** 2025-02-07

**Authors:** Zhichao Yang, Zonghai Yao, Mahbuba Tasmin, Parth Vashisht, Won Seok Jang, Feiyun Ouyang, Beining Wang, David McManus, Dan Berlowitz, Hong Yu

**Affiliations:** 1 College of Information and Computer Science University of Massachusetts Amherst Amherst, MA United States; 2 Miner School of Computer & Information Sciences University of Massachusetts Lowell Lowell, MA United States; 3 Shanghai Medical College Fudan University Shanghai China; 4 Department of Medicine University of Massachusetts Chan Medical School Worcester, MA United States; 5 Department of Public Health University of Massachusetts Lowell Lowell, MA United States; 6 Center for Biomedical and Health Research in Data Sciences University of Massachusetts Lowell Lowell, MA United States; 7 Center for Healthcare Organization and Implementation Research VA Bedford Health Care System Bedford, MA United States

**Keywords:** artificial intelligence, natural language processing, large language model, LLM, ChatGPT, GPT, GPT-4V, USMLE, Medical License Exam, medical image interpretation, United States Medical Licensing Examination, NLP

## Abstract

**Background:**

Recent advancements in artificial intelligence, such as GPT-3.5 Turbo (OpenAI) and GPT-4, have demonstrated significant potential by achieving good scores on text-only United States Medical Licensing Examination (USMLE) exams and effectively answering questions from physicians. However, the ability of these models to interpret medical images remains underexplored.

**Objective:**

This study aimed to comprehensively evaluate the performance, interpretability, and limitations of GPT-3.5 Turbo, GPT-4, and its successor, GPT-4 Vision (GPT-4V), specifically focusing on GPT-4V’s newly introduced image-understanding feature. By assessing the models on medical licensing examination questions that require image interpretation, we sought to highlight the strengths and weaknesses of GPT-4V in handling complex multimodal clinical information, thereby exposing hidden flaws and providing insights into its readiness for integration into clinical settings.

**Methods:**

This cross-sectional study tested GPT-4V, GPT-4, and ChatGPT-3.5 Turbo on a total of 227 multiple-choice questions with images from USMLE Step 1 (n=19), Step 2 clinical knowledge (n=14), Step 3 (n=18), the Diagnostic Radiology Qualifying Core Exam (DRQCE) (n=26), and AMBOSS question banks (n=150). AMBOSS provided expert-written hints and question difficulty levels. GPT-4V’s accuracy was compared with 2 state-of-the-art large language models, GPT-3.5 Turbo and GPT-4. The quality of the explanations was evaluated by choosing human preference between an explanation by GPT-4V (without hint), an explanation by an expert, or a tie, using 3 qualitative metrics: comprehensive explanation, question information, and image interpretation. To better understand GPT-4V’s explanation ability, we modified a patient case report to resemble a typical “curbside consultation” between physicians.

**Results:**

For questions with images, GPT-4V achieved an accuracy of 84.2%, 85.7%, 88.9%, and 73.1% in Step 1, Step 2 clinical knowledge, Step 3 of USMLE, and DRQCE, respectively. It outperformed GPT-3.5 Turbo (42.1%, 50%, 50%, 19.2%) and GPT-4 (63.2%, 64.3%, 66.7%, 26.9%). When GPT-4V answered correctly, its explanations were nearly as good as those provided by domain experts from AMBOSS. However, incorrect answers often had poor explanation quality: 18.2% (10/55) contained inaccurate text, 45.5% (25/55) had inference errors, and 76.3% (42/55) demonstrated image misunderstandings. With human expert assistance, GPT-4V reduced errors by an average of 40% (22/55). GPT-4V accuracy improved with hints, maintaining stable performance across difficulty levels, while medical student performance declined as difficulty increased. In a simulated curbside consultation scenario, GPT-4V required multiple specific prompts to interpret complex case data accurately.

**Conclusions:**

GPT-4V achieved high accuracy on multiple-choice questions with images, highlighting its potential in medical assessments. However, significant shortcomings were observed in the quality of explanations when questions were answered incorrectly, particularly in the interpretation of images, which could not be efficiently resolved through expert interaction. These findings reveal hidden flaws in the image interpretation capabilities of GPT-4V, underscoring the need for more comprehensive evaluations beyond multiple-choice questions before integrating GPT-4V into clinical settings.

## Introduction

Using computers to help make clinical diagnoses and guide treatments has been a goal of artificial intelligence (AI) since its inception [1]. The adoption of electronic health record systems by hospitals in the United States has resulted in an unprecedented amount of digital data associated with patient encounters. Computer-assisted clinical diagnostic support systems (CDSSs) endeavor to enhance clinicians’ decisions with patient information and clinical knowledge [2]. There is burgeoning interest in CDSS for enhanced imaging [3] in various disciplines such as breast cancer detection [4], COVID detection [5], diagnosing congenital cataracts [6], and hidden fracture location [7]. For a decision to be trustworthy for clinicians, CDSS should not only make the prediction but also provide accurate explanations [8-10]. However, most previous imaging CDSSs only highlight areas deemed significant by AI [11-14], providing limited insight into the explanation of the diagnosis [15].

Recent advances in large language models (LLMs) have encouraged much discussion in health care. State-of-the-art LLMs include GPT-3.5 Turbo, a chatbot released by OpenAI in October 2022, and its successor, GPT-4, released in March 2023. The success of GPT-3.5 Turbo and GPT-4 is attributed to their conversational ability and their performance, which have approached or matched human-level competence in cognitive tasks, spanning various domains including medicine [16]. Both GPT-3.5 Turbo and GPT-4 have achieved commendable results in the United States Medical Licensing Examination (USMLE), leading to discussions about the readiness of LLM applications for integration into clinical [17-19] and educational [20-22] environments.

One limitation of GPT-3.5 Turbo and GPT-4 is that they can only read and generate text and are unable to process other data modalities, such as images. This limitation, known as “single modality,” is a common issue among many LLMs [23]. Advancements in multimodal LLMs promise enhanced capabilities and integration with diverse data sources [24-26]. OpenAI’s GPT-4V is a state-of-the-art multimodal LLM equipped with image processing and understanding ability [27]. However, the ability of GPT-4V to answer medical questions with images with explanations has not been comprehensively evaluated. In this study, we aimed to expose hidden flaws in GPT-4V’s ability to interpret clinical images by thoroughly evaluating its performance on medical licensing examination questions involving image interpretation. For GPT-4V to be useful to medical professionals, it should not only provide correct responses but also offer accurate explanations for its reasoning, especially in complex multimodal clinical scenarios [28].

## Methods

### Overview

**Figure 1:**
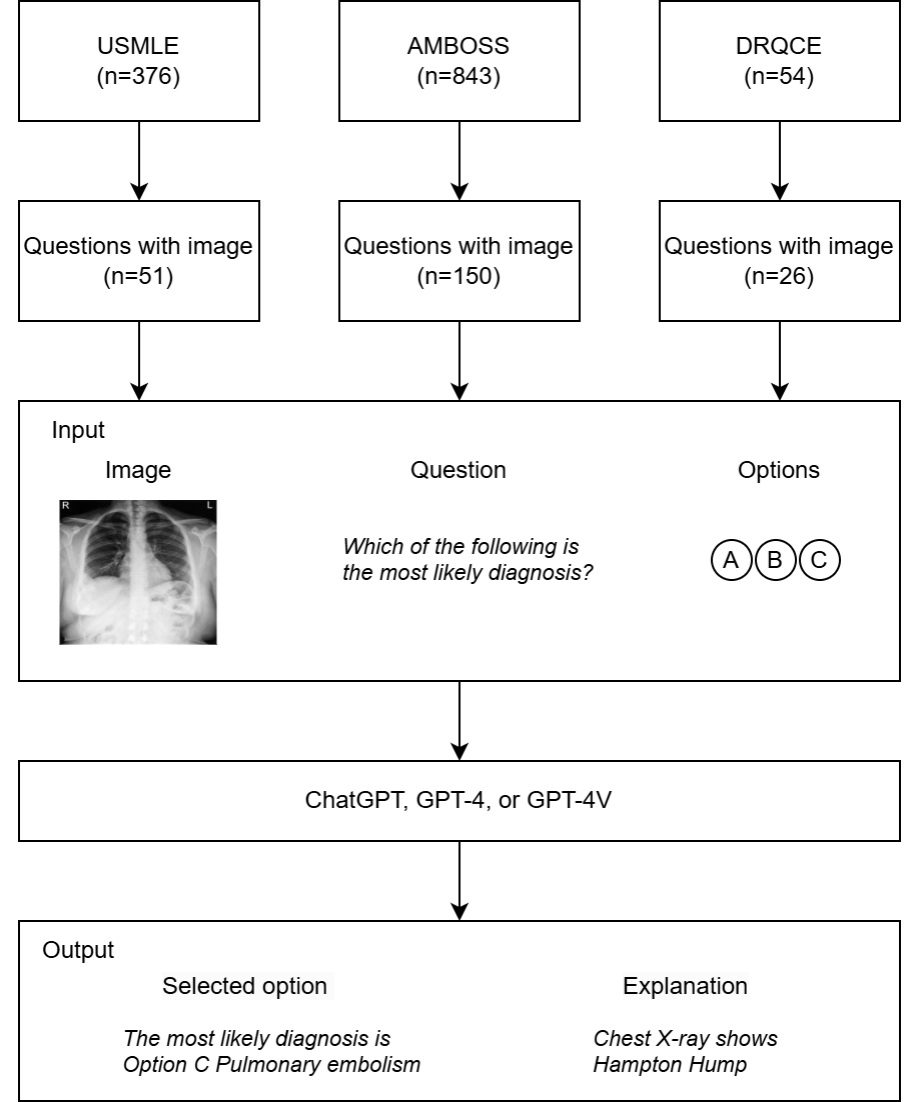
A summary of the image question selection process and prompt to large language models.

This cross-sectional study aimed to expose the hidden flaws of GPT-4V in clinical image interpretation by comparing the performance between GPT-4V, GPT-4, and GPT-3.5 Turbo in answering medical licensing examination questions. This study also investigates the quality of GPT-4V explanation in answering these questions. The overview of the study is shown in Figure 1. This study was conducted in October 2023.

### Ethical Considerations

The requirement for ethical approval and informed consent was waived by the institutional review board at the VA Bedford Health Care System because no patient data were used. The experiments were performed in accordance with the Declaration of Helsinki.

### Medical Exams and a Patient Case Report Collection

We obtained study questions from 3 sources. USMLE consists of 3 steps required to obtain a medical license in the United States. USMLE assesses a physician’s ability to apply knowledge, concepts, and principles, which is critical to both health and disease management and is the foundation for safe, efficient patient care. Step 1 assesses foundational scientific concepts essential for medical practice, Step 2 clinical knowledge (CK) evaluates the application of clinical science for supervised patient care, and Step 3 tests the medical knowledge required for unsupervised practice. Step 1, Step 2 CK, and Step 3 of the USMLE sample exam released from the National Board of Medical Examiners consist of 119, 120, and 137 questions respectively. We accessed these questions from publicly available links [29]. Each question contained multiple options to choose from. We then selected all questions with images, resulting in 19, 14, and 18 questions from Step 1, Step 2 CK, and Step 3. Medical subdomains include but are not limited to radiology, dermatology, orthopedics, ophthalmology, cardiology, and general surgery.

The sample exam only included limited questions with images. Thus, we further collected similar questions from AMBOSS, a widely used question bank for medical students, which provides students’ performance on the exam. The performance enabled us to assess the comparative effectiveness of the model. For each question, AMBOSS associated an expert-written hint to tip the student to answer the question and a difficulty level that ranges from 1-5. Levels 1, 2, 3, 4, and 5 represent the easiest 20%, 20%-50%, 50%-80%, 80%-95%, and 95%-100% of questions respectively [30]. Hints are designed to guide students to the correct answer. They are typically formatted as a short paragraph that describes the image. We manually checked that no hint had disclosed the answer directly. In addition to the gold standard choice, each answer is associated with a detailed explanation by AMBOSS. They were developed through an internal peer-review process involving more than 50 physicians who achieved high scores in the exam. We used a commercial license to access the questions. Since AMBOSS is not publicly available and its licensing terms restrict the automatic website scraping of its proprietary content, they are not in the CommonCrawl data set used to train GPTs [31]. We randomly selected and manually downloaded 10 questions from each of the 5 difficulty levels. We repeated this process for Step 1, Step 2 CK, and Step 3. This resulted in a total number of 150 questions.

In addition, we collected questions from the Diagnostic Radiology Qualifying Core Exam (DRQCE) [32], which is an image-rich exam to evaluate a candidate’s foundational knowledge and clinical judgment across practice domains of diagnostic radiology, which is offered after 36 months of residency training. Since DRQCE is proprietary, we used a commercial license to access the 26 questions with images of 54 questions in the preparation exam offered by the American Board of Radiology. In total, we had 227 questions with images from the 3 aforementioned sources.

To illustrate GPT-4V’s potential as an imaging diagnostic support tool and further expose its limitations, we used part of a patient case report [33] to resemble a typical “curbside consultation” between medical professionals [34]. In this case, the patient’s admission info, such as history of present illness, labs, and images of the case report will be presented to both a physician and GPT-4V. The physician can then work with GPT-4V through question answering, for example, by asking GPT-4V to help interpret images, for the final clinical diagnosis.

### How to Answer Image Questions Using GPT-4V Prompts

GPT-4V took image and text data as inputs to generate textual outputs. Given that input format (prompt) played a key role in optimizing model performance, we followed the standard prompting guidelines of the visual question-answering task [35]. Specifically, we prompted GPT-4V by first adding the image, then appending context (ie, patient information) and questions, and finally providing multiple-choice options, each separated by a new line. An example user prompt and GPT-4V response are shown in Figure S1 in Multimedia Appendix 1. When multiple subimages existed in the image, we uploaded multiple subimages to GPT-4V. We did not append a hint to the end of the question, unless other specified. The response consists of the selected option as an answer, supported by a textual explanation to substantiate the selected decision. When using GPT-3.5 Turbo and GPT-4 models that cannot handle image data, images were omitted from the prompt. These models were accessed through OpenAI application programming interfaces. Responses were collected from the September 25, 2023, version of models.

### Evaluation Metrics

For answer accuracy, we evaluated the model’s performance by comparing the model’s choice with the correct choice provided by the exam board or question bank website. We defined accuracy as the ratio of the number of correct choices to the total number of questions.

We also evaluated the quality of the explanation by preference from 3 health care professionals (1 medical doctor with 35 years of experience in internal medicine, 1 registered ward nurse with 2 years of experience, and 1 third-year medical school student). For each question from the AMBOSS data set (n=150), we first asked the health care professionals to choose their preference between an explanation by GPT-4V (without hint), an explanation by an expert, or a tie without knowing the correctness of GPT-4V’s answers. The exclusion of correctness is to avoid bias in their preference of explanations. In addition, the source of the explanations was blinded to the health care professionals, ensuring that their judgments were not influenced by knowing whether an explanation came from GPT-4V or an expert.

In addition, we also asked health care professionals to evaluate the GPT-4V explanation from a sufficient and comprehensive perspective [36,37]. They determined if the information exists in the explanation, that consists of (1) image interpretation: GPT-4V tried to interpret the image in the explanation, and such interpretation is sufficient to support its choice; (2) question information: explanations contained information related to the textual context (ie, patient information) of the question, and such information was essential for GPT-4V’s choice; (3) comprehensive explanation: the explanation included comprehensive reasoning for all possible evidence (eg, symptoms, lab results) that leads to the final answer.

Finally, for each question answered incorrectly, we asked health care professionals to check if the explanation contained any errors that consisted of (1) image misunderstanding (if the sentence in the explanation showed an incorrect interpretation of the image; eg, GPT-4V said that a bone in the image was for the hand, but it was in fact the foot); (2) text hallucination (if the sentence in the explanation contained made-up information [38]; eg, claiming Saxenda was insulin); (3) reasoning error (if the sentence did not properly infer knowledge in either image or text to an answer; eg, GPT-4V reasoned that a patient took a trip within the last 3 months and therefore diagnosed the patient as having Chagas disease, despite the clinical knowledge that Chagas disease usually develops 10-20 years after infection); or (4) nonmedical error (GPT is known to struggle with tasks requiring precise spatial localization, such as identifying chess positions on the board [27]).

In this study, we asked an internal medicine doctor with 35 years of experience to articulate a detailed rating guideline above. Our study has shown that the medical student and nurse, both of whom participated independently, agreed with the doctor’s ratings of 95% and 86%, respectively. This high agreement ratio underscores the effectiveness of the standardized guidelines in ensuring consistent evaluation across varying levels of expertise.

### Statistical Analysis

Chi-square tests and pairwise comparisons with Bonferroni corrections were used for the performance metrics of GPT-3.5 Turbo, GPT-4, and GPT-4V on visual question answering exams. GPT-4V’s accuracies on the AMBOSS data set were compared between different difficulties using unpaired chi-square tests with a significance level of 0.05. All analysis was conducted in Python software (version 3.10.11; Python Software Foundation).

## Results

### Overall Answer Accuracy

For all questions in the USMLE sample exam (including ones without images), GPT-4V achieved an accuracy of 88.2%, 90.8%, and 92.7% among Step 1, Step 2 CK, and Step 3 of USMLE questions, respectively. In comparison, GPT-3.5 Turbo and GPT-4 achieved an accuracy of 55.1% and 81.5% in Step 1, 59.1% and 80.8% in Step 2 CK, and 60.9% and 88.3% in Step 3, respectively (Table 1). GPT-4V outperformed GPT-4 and GPT-3.5 Turbo by 11.3% (95% CI 11.5%-11.1%; *P*<.001) and 32% (95% CI 32.3%-31.7%; *P*<.001). The score of GPT-4V passes the standard for the USMLE (about 60%). The performance of GPT-4V across different subdomains is shown in Table S1 in Multimedia Appendix 1.

**Table 1:** Performance of GPT-3.5 Turbo, GPT-4, and GPT-4V on a USMLE sample exam from the National Board of Medical Examiners without hints.

Exam name and agents		Performance	
		Questions with image, n (%)	All questions, n (%)
**USMLE^a^ sample exam-Step 1^b^**			
	Sample size	19	119
	GPT-3.5 Turbo	8 (42.1)	66 (55.1)
	GPT-4	12 (63.2)	97 (81.5)
	GPT-4V	16 (84.2)	105 (88.2)
**USMLE sample exam-Step 2 clinical knowledge^c^**			
	Sample size	14	120
	GPT-3.5 Turbo	7 (50)	71 (59.1)
	GPT-4	9 (64.3)	97 (80.8)
	GPT-4V	12 (85.7)	109 (90.8)
**USMLE sample exam-Step 3^d^**			
	Sample size	18	137
	GPT-3.5 Turbo	9 (50)	73 (60.9)
	GPT-4	12 (66.7)	121 (88.3)
	GPT-4V	16 (88.9)	127 (92.7)
**DRQCE^e^ sample exam^f^**			
	Sample size	26	54
	GPT-3.5 Turbo	5 (19.2)	31 (57.4)
	GPT-4	7 (26.9)	35 (64.8)
	GPT-4V	19 (73.1)	48 (88.9)

^a^USMLE: United States Medical Licensing Examination.

^b^19 questions with images and 119 questions in total in Step 1.

^c^14 questions with images and 120 questions in total in Step 2 CK.

^d^There were 18 questions with images and 137 questions in total in Step 3.

^e^DRQCE: Diagnostic Radiology Qualifying Core Exam.

^f^There were 26 questions with images and 54 questions in total in DRQCE.

For questions with images, GPT-4V achieved an accuracy of 84.2%, 85.7%, and 88.9% in Step 1, Step 2 CK, and Step 3 of USMLE questions, respectively. It outperformed GPT-3.5 Turbo and GPT-4 by 42.1% (8/19; 95% CI 36.8%-47.4%; *P*<.001) and 21.1% (4/19; 95% CI 7.8-34.2%; *P*=0.01) in Step 1, 35.7% (5/14; 95% CI 3.1%-39.7%; *P*=.03) and 21.4% (3/14; 95% CI 4.7%-38.1%; *P*=.02) in Step 2 CK, 38.9% (7/18; 95% CI 32.2%-45.7%; *P*<.001) and 22.2% (4/18; 95% CI 5.5%-38.9%; *P*=.02) in Step 3, respectively. Similarly, GPT-4V achieved an accuracy of 73.1%, outperforming GPT-3.5 Turbo by 53.9% (14/26; 95% CI 41.6%-66.2%; *P*<.001) and GPT-4 by 46.2% (12/26; 95% CI 29.8%-62.5%; *P*<.001) in DRQCE (Table 1). This highlights the superior ability of GPT-4V to interpret clinical images compared with earlier versions.

### Impact of Difficulty Level and Use of Hints

When asking GPT-4V questions without a hint, it achieved an accuracy of 60%, 64%, and 66% for AMBOSS Step 1, Step 2 CK, and Step 3, respectively (Table 2). GPT-4V was in the 72nd, 76th, and 80th percentile with AMBOSS users who were preparing for Step 1, Step 2 CK, and Step 3, respectively. When asking GPT-4V questions with a hint, it achieved an accuracy of 84%, 86%, and 88% for AMBOSS Step 1, Step 2 CK, and Step 3, respectively. Figure S2 in Multimedia Appendix 1 is an example where GPT-4V switched the answer from incorrect to correct when a hint was provided. GPT-4V predictions on the entire AMBOSS data set with images are reported in Table S2 in Multimedia Appendix 1 (n=646). Conclusions drawn from automatic evaluation align with our findings presented in Table 2 (n=150).

**Table 2:** Performance of GPT-4V on AMBOSS.

AMBOSS steps and hint availability			GPT-4V accuracy on AMBOSS, %										
			Overall (n=50)		1 (n=10)		2 (n=10)		3 (n=10)		4 (n=10)		5 (n=10)
**Step 1**													
	Without hint	60		70		70		30		70		60	
	Expert hint	84		80		80		80		90		90	
**Step 2 clinical knowledge**													
	Without hint	64		80		70		70		50		50	
	Expert hint	86		100		90		100		70		70	
**Step 3**													
	Without hint	66		80		90		60		50		50	
	Expert hint	88		90		90		90		90		80	

**Figure 2:**
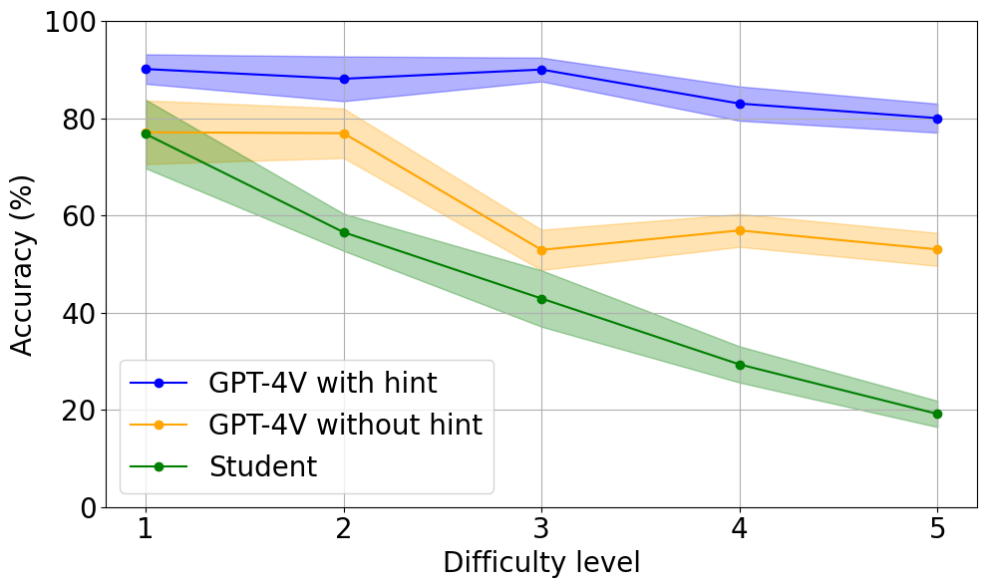
Performance of GPT-4V and students on 150 AMBOSS questions with different difficulty levels.

Figure 2 shows a decreasing trend in GPT-4V’s performance in the AMBOSS data set when the difficulty of questions increased (*P*=.04) without a hint. However, with the hint, the performance of GPT-4V plateaued across 5 difficulty levels. Importantly, the accuracies of both GPT-4V, with or without a hint, in general, outperformed the accuracies of medical students, and the gap between the performance of GPT-4V and medical students increased when the difficulty increased. On the most difficult questions, GPT-4V with hint outperformed medical students by 60% (18/30, 95% CI 56.8%-63.1%; *P*<.001), and GPT-4V without hint outperformed medical students by 26.7% (8/30, 95% CI 24.2%-29.3%; *P*<.001). The findings show that while GPT-4V outperforms medical students in accuracy, its performance is largely dependent on context-based hints, reflecting a fundamental flaw in image reasoning.

### Quality of Explanation

We evaluated the user’s preference among GPT-4V-generated explanations and expert-generated explanations. When GPT-4V answered incorrectly, our results show that health care professionals overwhelmingly preferred expert explanations as shown in Table 3. In total, 47 preferred experts and 0 preferred GPT-4V. When GPT-4V answered correctly, the quality of GPT-4V-generated explanations was close to expert-generated explanations: out of 95 votes, 19 preferred experts, 15 preferred GPT-4V, and 61 preferred either. The preference for expert explanations in incorrect answers highlights key weaknesses in GPT-4V’s ability to interpret clinical images accurately and offer dependable reasoning.

**Table 3:** Health care professionals preferred explanations for 150 AMBOSS questions.

AMBOSS steps and correctness of GPT-4V (without hint) responses		Health care professionals’ preference		
		Prefer expert	Ties	Prefer GPT-4V
**Step 1**				
	Correct	4	23	3
	Incorrect	16	4	0
**Step 2 clinical knowledge**				
	Correct	10	15	7
	Incorrect	18	0	0
**Step 3**				
	Correct	5	23	5
	Incorrect	13	4	0

We further evaluated the quality of the GPT-4V generated explanation by verifying if the explanation includes image and question text interpretation in Table S3 in Multimedia Appendix 1. When examining the 95 correct answers, 84.2% (n=80) of the responses contained an interpretation of the image, while 96.8% (n=92) aptly captured the information presented in the question. On the other hand, for the 55 incorrect answers, 92.8% (n=51) interpreted the image, and 89.1% (n=49) depicted the question’s details. In terms of comprehensiveness, GPT-4V offered a comprehensive explanation in 79% (n=75) of correct responses. In contrast, only 7.2% (n=4) of the wrong responses had a comprehensive explanation that led to the GPT-4V’s choice.

We also evaluated the explanations of incorrect responses by GPT-4V image and grouped them into 4 categories, that are image misunderstanding, text hallucination, reasoning error, and nonmedical error. Among GPT-4V responses with wrong answers (n=55), we found that 76.3% (n=42) of responses included misunderstanding of the image, 45.5% (n=25) of responses included logic error, 18.2% (n=10) of responses included text hallucination, and no responses included nonmedical errors.

### A Case Study of Curbside Consultation

We present a clinical case study involving a 45-year-old woman with hypertension and altered mental status. As shown in Figure S3 in Multimedia Appendix 1, a collaborative design of GPT-4V allows communication between GPT-4V and physicians. In this scenario, when asked to interpret a CT scan, GPT-4V initially provided an irrelevant answer. GPT-4V needed 5 additional physician-guided prompts to list potential diagnoses, including primary aldosteronism, hypertension, and Cushing’s syndrome. For instance, when the physician specifically prompted, “If I suspect Cushing’s syndrome due to ectopic ACTH secretion, what would be the next steps to evaluate this patient to determine the source of the hormonal abnormality?” and pointed to a specific area on the CT scan, GPT-4V was then able to respond correctly. This interaction indicates that GPT-4V struggles to autonomously interpret medical images, requiring continuous and specific prompts for accurate interpretation, which underscores its flaws in independent image reasoning.

## Discussion

### Principal Findings

Recent advancements in medical question-answering systems have leveraged domain-specific transformer models. Early models such as PubMedBERT [39] with 100 million parameters score around 38.3% in USMLE. The introduction of larger models marked a substantial improvement. JMLR [40] with 13 billion parameters, Med-Palm [41] with 540 billion parameters, and GPT-4 achieves 62.5%, 86.2%, and 90.2% respectively. However, previous works only tested these models on text-only questions without images [20,42-44] or questions in non-English languages [45,46]. Unlike previous works that focus primarily on accuracy [47,48], we emphasize explanation quality as a crucial metric for assessing the model’s clinical applicability. In particular, we evaluated GPT-4V’s ability to interpret medical images (a new feature) to highlight hidden flaws in clinical image interpretation.

We found that GPT-4V outperformed both GPT-3.5 Turbo and GPT-4 (Table 1). When evaluating all questions in the USMLE sample exam, GPT-4V achieved an accuracy of 90.7% outperforming GPT-3.5 Turbo (58.5%) and GPT-4 (83.8%). In comparison, medical students can pass the USMLE exam with more than 60% accuracy, indicating that the GPT-4V performed at a level similar to or above a medical student in the final year of study. The accuracy of GPT-4V highlights its grasp over biomedical and clinical sciences, essential for medical practice, and showcases its ability in patient management and problem-solving skills [49]. Other studies further demonstrated the potential for clinical routines, such as summarizing radiology reports [50] and differential diagnosis [51,52].

For medical exam questions with images, we found that GPT-4V achieved an accuracy of 62%, which was equivalent to the 70th-80th percentile with AMBOSS medical students. This finding indicates that GPT-4V has the capability to integrate information from both text and images to answer questions, making it a promising tool for answering clinical questions based on images. However, our evaluation also reveals hidden flaws in its image interpretation, particularly in its inconsistency and the need for extensive context to provide accurate answers.

Another important finding is that GPT-4V significantly outperformed medical students for questions considered difficult for the students. Specifically, our results, as shown in Figure 2, show that while medical students’ performance linearly decreased when the difficulty of questions increased, GPT-4V’s performance stayed relatively stable. When expert hints were provided, GPT-4V’s performance stayed plateau among questions in all difficult levels. This consistent performance indicates that GPT-4V effectively addresses questions that medical students find challenging. Its advanced capabilities suggest potential as an educational assistant, particularly for complex topics. Under the supervision of teachers’ hints, medical students could benefit from its advanced capabilities to understand and analyze complex medical questions.

There may be multiple factors that contribute to GPT-4V’s performance on difficult questions. Instrument methods (eg, item response theory [53]) have been typically used for the construction and evaluation of measurement scales and tests. For example, item response theory uses a statistical model that links an individual person’s responses to individual test items (questions on a test) to the person’s ability to correctly respond to the items and the items’ features. Therefore, medical examination test sets have been specifically selected and tailored to medical students’ performance with the intended distribution where the performance decreases when the difficulty level increases. Although more evaluation is needed to draw the conclusion that GPT-4V substantially outperformed medical students in difficult questions, our results at least show that GPT-4V performed differently.

On the other hand, we found that GPT-4V’s performance was inconsistent among different medical subdomains. As shown in Table S1 in Multimedia Appendix 1, GPT-4V achieved high accuracy on subdomains such as immunology (5/5, 100%), otolaryngology (6/6, 100%), and pulmonology (6/8, 75%), and low accuracy on others such as anatomy (1/4, 25%), emergency medicine (1/4, 25%), and pathology (5/10, 50%). This suggests that while GPT-4V shows potential in some specialties or subdomains, it may require further development to be reliable across the board. The uneven performance highlights the need for tailored approaches to enhancing the model’s capabilities where it falls short.

Another advantage of GPT-4V is its ability to explain its image content. Previous studies have shown limited use of current CDSS as most of them offered limited decision explanations and thus gained limited trust among physicians (unlike their colleagues) [54-57]. In contrast, GPT-4V has the potential to improve the effectiveness and credibility of CDSS by providing explanations preferred by experts. As our results indicate, the quality of explanations generated by GPT-4V, when answering correctly, is close to that of expert-generated explanations. Although in more complex scenarios (such as in our curbside consult setting), GPT-4V currently requires continuous highly specialized guidance, which temporarily prevents it from enhancing physician work efficiency, this feature still has the potential to encourage physicians to adopt and use GPT-4V more confidently and broadly.

In terms of explanation quality, we found that more than 80% of responses from GPT-4V provided an interpretation of the image, regardless of whether the responses were correct or not. This suggests that GPT-4V consistently takes into account the image while generating responses. Figure S1 in Multimedia Appendix 1 illustrates an example of a high-quality explanation that uses images to answer a hard question. In this example, more than 70% of students answered incorrectly on the first try, because both bacterial pneumonia and pulmonary embolism may involve symptoms such as cough. To differentiate them, GPT-4V correctly interpreted the x-ray with a radiologic sign of Hampton hump, which further increased the suspicion of pulmonary infarction rather than pneumonia [58]. To show the need for an x-ray as mentioned in the explanation, we removed the image from the input, and GPT-4V switched the answer to bacterial pneumonia while also acknowledging the possibility of pulmonary infarction. This change in response demonstrated the high quality of the GPT-4V explanation, as its explanation about x-rays was not fictional and it truly needed the x-ray to answer this question.

On the other hand, we found that the quality of generated explanations was poor when GPT-4V answered incorrectly. Manual analyses by health care professionals concluded that image misunderstanding was the primary reason why GPT-4V answered incorrectly. Out of 55 wrong responses, 42 (76.3%) were due to misunderstanding of the image. In comparison, only 18.2% (10/55) of the mistakes were attributed to text misinterpretation. Clearly, GPT-4V’s proficiency in processing images was considerably lagging behind its text-handling capability. This gap in capability suggests that GPT-4V’s advancements in image understanding remain nascent and require significant refinement to align with its text analysis capabilities. To circumvent its image interpretation issue, we additionally prompted GPT-4V with a short hint that described the image. We found that 40% (22/55) of responses switched to the correct answer. One potential future direction involves strengthening GPT-4V's domain-specific knowledge by integrating extensive clinical datasets into its training. For example, employing domain-adaptive pretraining methods—such as those used in MEDITRON [59], which leverages medical guidelines and specialized clinical corpora—could significantly improve the model’s understanding of medical concepts, leading to more precise and contextually relevant explanations. In addition, incorporating retrieval-augmented generation based on domain-specific corpora [40] would enable the model to access and retrieve pertinent clinical information during inference, grounding its explanations in verified data. This could improve factual accuracy and reduce the likelihood of incorrect or unsupported responses. Together, these strategies aim to bolster the model's capacity to provide high-quality, accurate explanations, thereby enhancing its overall reliability and usefulness in clinical applications.

Creating these image-related hints requires clinical expertise, limiting the use of GPT-4V as a CDSS. In our case study, when GPT-4V delivered an irrelevant response, the physician needed to come up with correct hints for GPT-4V. These findings reveal a key limitation: GPT-4V’s reliance on external guidance from experts to interpret complex image content effectively, thereby exposing its inability to operate independently in clinical scenarios. Efforts improving GPT-4V on images include multimodal LLMs with reinforcement learning from human feedback to align the outputs of LLMs with physicians’ intentions and expectations. This alignment is critical not only for enhancing the accuracy and relevance of the responses but also for integrating GPT-4V seamlessly into clinical environments where time is of the essence [60].

Another significant drawback of GPT-4V involved its tendency to produce factually inaccurate responses, a problem often referred to as the hallucination effect, which is prevalent among many LLMs such as GPT-4V [38]. We found that more than 18% of GPT-4V explanations contain hallucinations, potentially misleading or distracting physicians, particularly the less experienced medical students and residents. This finding emphasizes the need for robust evaluation and correction mechanisms to minimize hallucinations, which are critical to ensure GPT-4V’s reliability and safety in clinical practice. One future direction is to integrate GPT-4V and a probabilistic model with CI and citations from credible sources to show the reliability of the response [40,61,62]. The confidence score could also help prioritize the list of differential diagnoses, making it clearer to the physician which conditions are more probable. Thereby reducing the risk of confusion and enhancing the reliability of the CDSS response when additional physician review is warranted [15].

### Limitations

This study has several limitations. First, our findings are constrained in their applicability due to the modest sample size. We gathered 227 questions from a total of 28 subdomains or specialties that included images, which might not comprehensively represent all medical disciplines. The small number of questions in each subdomain may not be sufficient to conclude that GPT-4V’s performance is inconsistent between medical subdomains. Second, the exams used to test GPT-4V are written in English. Future work could explore other languages. Third, the models used for evaluation were from September 2023, and frontier models may have evolved since then, potentially impacting the results. Fourth, we cannot guarantee that OpenAI strictly adhered to licensing terms when determining which content was included or excluded from their training sets; therefore, even though AMBOSS is not publicly available and its licensing terms restrict the automatic website scraping of its proprietary content, GPT may have already seen the data during training, potentially impacting the results. Finally, while GPT-4V has demonstrated proficiency in medical license examination, its CDSS ability remains untested. Future work could explore continued training GPT-4V in the medical domain for better CDSS integration. Medical exams provide options, but such options would rarely be provided by physicians during CDSS. Our study highlights the inherent limitations in GPT-4V’s image interpretation abilities, particularly without expert guidance. We showed that GPT-4V can reduce errors with expert hints, but in more realistic clinical environments, it required continuous highly specialized guidance to make partially correct diagnoses and subsequent examination recommendations, revealing limitations in its autonomous decision-making capabilities. Therefore, more cases with clinician questions should be explored to confirm our findings before clinical integration. Extrapolating the efficacy of GPT-4V to broader clinical applications requires appropriate benchmarks and further research.

Regarding ethical considerations, deploying AI systems for medical advice poses significant ethical implications, especially in medical education and clinical decision-making. Incorrect AI-generated explanations risk disseminating misinformation that could misguide medical professionals, impacting patient safety and treatment outcomes. This is particularly concerning when AI is used in training settings, as it could shape the decision-making abilities of future healthcare providers in potentially harmful ways. Integrating AI into clinical workflows also raises broader societal concerns. While AI has the potential to enhance healthcare efficiency, it could alter patient care dynamics and physician roles. Overreliance on AI may reduce direct physician-patient communication, eroding trust and undermining the relationship-building essential for effective care. Physicians might also become too dependent on AI, potentially compromising their clinical judgment and their ability to critically assess AI-generated insights. Thus, integrating AI in a manner that complements human expertise (supporting rather than replacing health care providers) is vital. Moreover, current benchmarks, including the one in our study, do not fully assess an AI’s capabilities for real-world clinical decision-making. Although some LLMs perform well on benchmarks, they lack the comprehensive clinical skills and nuanced understanding required to navigate complex medical scenarios effectively. Viewing these AI models as tools that assist rather than replace clinicians is crucial to ensuring their safe and beneficial use in health care. A responsible approach is needed when deploying AI for medical advice, one that ensures ethical standards are maintained. Issues such as privacy, bias, and the broader implications of AI in society must guide the development and implementation of these systems. By enhancing data diversity, ensuring privacy, and fostering a transparent understanding of AI’s role, we can work toward ethical advancements in health care that enhance outcomes without compromising human oversight or patient trust. Future work should focus on developing AI technologies that are fully aligned with health care professionals, maintaining a collaborative and ethically sound approach to their integration.

### Conclusion

In this study, GPT-4V demonstrated remarkable overall accuracy on the medical licensing examination and provided high-quality explanations when correct. The evaluation of questions with images (a relatively novel feature for GPT models) allowed us to expose hidden flaws in GPT-4V’s image interpretation abilities, offering a unique insight into its strengths and weaknesses. Its performance on image-related questions ranged from 60% to 88%, while physician misdiagnosis rates can be as high as 40% [63,64]. GPT-4V substantially outperformed medical students on difficult questions, but we observed severe issues in its explanations and reasoning, including hallucinations, errors, and misinterpretations. These findings reveal significant challenges in GPT-4V’s ability to independently interpret and reason through complex image-based questions, which is crucial for clinical applications. Despite its strong performance on multiple-choice questions, GPT-4V may still encounter comprehension or explanation errors. When assisted by human experts, GPT-4V reduced some errors with image-related hints. However, in realistic curbside consult settings, continuous and highly specialized prompting was still required, making it time-consuming and limiting its utility as a clinical decision support system in real-world clinical practice. Table 4 lists the summary of key findings.

**Table 4:** Summary of key findings.

Metric	Findings
Accuracy of image-based questions	GPT-4V achieved 84.2% in Step 1, 85.7% in Step 2 CK^a^, 88.9% in Step 3, and 73.1% in DRQCE^b^, outperforming GPT-3.5 Turbo (42.1%, 50%, 50%, 19.2%) and GPT-4 (63.2%, 64.3%, 66.7%, 26.9%).
Explanation quality	When GPT-4V provided correct answers, its explanations were almost on par with those given by domain experts. However, for incorrect responses, the explanation quality was often lacking: 18.2% included inaccurate information, 45.5% involved inferencing mistakes, and 76.3% reflected misinterpretations of images.
The impact of human expert hints	There is a decreasing trend in GPT-4V’s performance in the AMBOSS dataset when the difficulty of questions increased) without hint. However, with the hint, the performance of GPT-4V plateaued.
Performance of GPT-4V on most difficult questions	GPT-4V with hints outperformed medical students by 60%, and GPT-4V without hints outperformed medical students by 26.7%.

^a^CK: clinical knowledge.

^b^DRQCE: Diagnostic Radiology Qualifying Core Exam.

Overall, our findings emphasize the need for a more comprehensive evaluation of GPT-4V’s multimodal capabilities, especially in clinical image interpretation, before considering its integration into clinical decision support systems. Future randomized clinical trials will help further verify the actual utility of GPT-4V and promote more extensive and profound integration of AI in the medical domain.

## Appendix

Multimedia Appendix 1 Supplementary figures and tables.

## Abbreviations

AI: artificial intelligence
CDSS: clinical diagnostic support system
CK: clinical knowledge
DRQCE: Diagnostic Radiology Qualifying Core Exam
LLMs: large language models
USMLE: United States Medical Licensing Examination

## Data Availability

The data sets generated during and/or analyzed during this study are available in the USMLE [29], AMBOSS [30], and DRQCE [32] repositories.
